# Preclinical testing of antiviral siRNA therapeutics delivered in lipid nanoparticles in animal models – a comprehensive review

**DOI:** 10.1007/s13346-025-01815-x

**Published:** 2025-02-25

**Authors:** Yusuf M. Idres, Adi Idris, Wenqing Gao

**Affiliations:** Centre for Immunology and Infection Control, School of Biomedical Sciences, Queensland University of Technology, Brisbane, QLD Australia

**Keywords:** siRNA, LNP, Antiviral, RNAi

## Abstract

The advent of RNA interference (RNAi) technology through the use of short-interfering RNAs (siRNAs) represents a paradigm shift in the fight against viral infections. siRNAs, with their ability to directly target and silence specific posttranscriptional genes, offer a novel mechanism of action distinct from that of traditional pharmacotherapeutics. This review delves into the growing field of siRNA therapeutics against viral infections, highlighting their critical role in contemporary antiviral strategies. Importantly, this review will solely focus on the use of lipid nanoparticles (LNPs) as the ideal antiviral siRNA delivery agent for use in vivo. We discuss the challenges of siRNA delivery and how LNPs have emerged as a pivotal solution to enhance antiviral efficacy. Specifically, this review focuses on work that have preclinically tested LNP formulated siRNA on virus infection animal models. Since the COVID-19 pandemic, we have witnessed a resurgence in the field of RNA-based therapies, including siRNAs against viruses including, SARS-CoV-2. Notably, the critical importance of LNPs as the ideal carrier for precious ‘RNA cargo’ can no longer be ignored with the advent of mRNA-LNP based COVID-19 vaccines. siRNA-based therapeutics represents an emerging class of anti-infective drugs with a foreseeable future as suitable antiviral agents.

**Graphical Abstract:**

**Figure:**
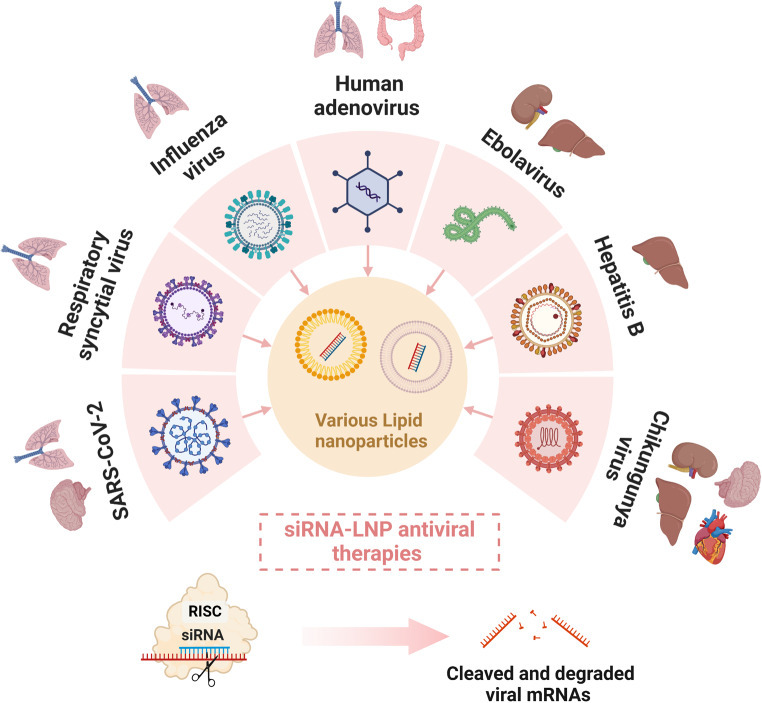

## Highlights

siRNAs can be harnessed as the next generation of oligonucleotide-based antiviral therapeutics.

Lipid nanoparticles (LNPs) overcome the challenges associated with the delivery and maintenance of antiviral siRNAs.

RNA interference (RNAi) as a programmable antiviral therapeutic is the next frontier in RNA medicine.

## Introduction

Since the discovery of RNA interference (RNAi) and evidence of RNAi in the form of short-interfering RNA (siRNA) in mammals [1], research and development in the siRNA therapies space have surged. siRNA therapeutics (20–25 bp long) leverage the RNAi pathway, a mechanism that cells use to regulate gene expression and defend against viral infections (see review by Vadhav et al. [2]). This RNAi pathway is initiated by a ribonuclease that processes long dsRNA molecules into shorter 20–25 nucleotide siRNAs. These siRNAs are then loaded into the RNA-induced silencing complex (RISC), with the Argonaute protein (AGO2) playing a pivotal role in unwinding the siRNA and retaining the guide strand. The guide strand of siRNA then directs RISC to complimentary mRNA sequences within the cytoplasm, followed by Watson-Crick base pairing. Once bound, the mRNA is cleaved and degraded, preventing the translation of proteins and effectively silencing gene expression.

Notably, nucleic acid synthesis technologies have allowed for the precise, rapid, and cost-effective production of siRNA molecules unlike small molecule drugs. This allows for greater scalability from the initial research and development stages [3]. Currently, there are five approved siRNA drugs (patisiran, givosiran, inclisiran, lumasirin and vurtisiran), with several others currently in late stages of phase III clinical trials [4]. Viruses present a challenge for therapeutic development as they are susceptible to high mutation rates, giving them the ability to evade host immune defence and develop resistance towards vaccines and conventional antivirals [5]. The recent COVID-19 pandemic was a testament to that. The development of new antiviral strategies, notably siRNA-based therapies, thereby becomes crucial. The mode of action for siRNAs is distinct from small molecule drugs, as it intervenes at the transcriptional mRNA level, as compared to the protein level, where protein drug targets may adopt spatial conformational changes. This also allows for targeting of viral mRNA; thus, siRNAs can be designed and used for different virally infected in vivo models. Such mechanisms have fast-tracked siRNA into clinical research, with some siRNAs already reaching the clinic (see FDA 2023 approvals [6]). Even with the advances in small-molecule drugs and vaccine development, the pace of developing new antiviral strategies only continue to increase in demand to match the rapid emergence and evolution of viruses. Therapeutic antiviral development can target any obligate stage of the viral life cycle, although the effectiveness may differ among viruses due to variations in their replication strategies and life cycle complexity. siRNA is programmable and modular in that it can be designed to target any viral mRNAs of essential genes critical to its lifecycle.

Despite the success of siRNA, delivery to target tissues and organs remains a challenge that still needs addressing due to rapid ribonuclease-mediated degradation, renal clearance, off-target effects, and sub-optimal release of siRNAs.

To maximise treatment efficacy, significant research has been dedicated to developing improved delivery platforms. Effective pharmacological use of siRNA requires a suitable ‘carrier’ that can deliver the nucleic acid cargo to the intended site of action. The role of these carriers are important, as they assemble the siRNA into supramolecular complexes that is essential to the functional properties of siRNA [7]. Viral vectors and non-viral vectors are the two major categories for nucleic acid delivery of siRNA. Viral-vector-based gene delivery systems has produced successful clinical data, but the effectiveness is limited by payload size constraints, risk for genomic integration, viral-induced immunogenicity, and poorer potential for scalability. Concurrent advances in the development of synthetic materials to encapsulate RNA payloads has since made its way into the clinic as a non-viral vector delivery platform. Notably, the use of lipid nanoparticles (LNPs) has demonstrated its success as a delivery platform for siRNA therapeutics [8], parallel to recent successes in mRNA-based COVID-19 vaccines encapsulated in LNPs [2]. LNPs are one of the leading delivery systems for oligonucleotides and has now established itself as one of the most clinically implicated non-viral gene delivery methods. Onpattro^®^ was the first RNAi therapeutic approved by the Food and Drug Administration (FDA) in 2018 [9]. Since then, LNP technology has elevated as the platform for gene editing, protein replacement and vaccine delivery. A testament to that, includes the role of LNPs in the development of several mRNA vaccines, such as Pfizer-BioNTech (BNT162b2) [10] and Moderna (mRNA-1273) [11]. LNPs generally are spherical in nature with a size of 100–400 nm, consisting of ionizable lipids, PEGylated lipids, helper lipids and cholesterol [12]. These give the structural and biological properties of LNPs and are adjusted in molar ratios according to the therapeutic context. Given the promise in siRNA as an antiviral agent, its therapeutic effectiveness is highly dependent on effective delivery. Therefore, we believe that LNPs are currently the most clinically advanced nonviral carriers for the delivery of siRNA. This review aims to explore the current antiviral approaches that utilise the promise of siRNA-based therapies using LNP technology as the key delivery vehicle. Importantly, we review all pre-clinical studies to date that have used LNP encapsulated antiviral siRNAs tested in virally infected animal models.

### RNAi

Depending on the RNA drug mechanism of action, it is important to consider how these mechanisms may influence drug delivery vehicle design. siRNA utilises endogenous enzymes within eukaryotic cells, such as the RISC to achieve gene silencing, thus do not require the delivery of large enzymes. Similarly, microRNAs (miRNAs) recruit the RISC to its complementary mRNA sequences, causing an RNA interference effect. As a result of this miRNA are also studied within virally infected animal models [13] and have certainly made its way into clinical trials [14]. Similarly, short hairpin RNAs (shRNA), are sequences that are continuously expressed within a DNA vector or plasmid through a promoter region to drive continuous expression. This makes it advantageous for long-term gene silencing, so may be suitable in the context of a chronic viral infection but has also been used in acute infections models in vivo. These hairpin structures are processed by the enzyme dicer into siRNA sequences. Chronic viral infections are often associated with a sustained type I interferon response [15]. The type I interferon response has been suggested to inhibit the RNAi pathway by inhibiting dicer, preventing the processing of the shRNA into siRNA [16].

While siRNA hold promises as therapeutic agents, its pathway to success has been fraught with challenges. Importantly, it has the ability to trigger unfavourable immune responses [17]. Endoribonucleases are the enzymes involved in RNA degradation via the cleavage of RNA phosphodiester bonds located at the 2’OH (hydroxyl) group. Therefore, chemical modifications can be essential in siRNA design (e.g., conjugation of 2’F fluorine or 2’O-Me methyl groups), rendering siRNAs less susceptible to the endoribonucleolytic cleavage at the 2’OH group, enhancing overall siRNA stability [18] and reduced unwanted immunostimulatory activity [19]. Whilst the cost of chemical consideration may be expensive, they remain a critical component of most clinically approved siRNA therapeutics and are necessary for achieving stability and efficacy in many experimental and clinical contexts. Ensuring that these siRNA molecules reach their intended target tissue or cells is also challenging. This is largely in part to due to poor stability during circulation and unfavourable pharmacokinetics leading to reduced biodistribution.

### siRNAs as a new class of antiviral drugs

Traditionally, antiviral drugs target viral enzymes and or structural proteins, identified through large drug screens or rational drug design. Small molecule drugs that target viruses have seen its share of success over the past several decades [20]. However, the potency and predictable efficacy of small molecule drugs can vary depending on the type of virus and its route of infection [21]. Moreover, the screening, chemical optimisation, small animal toxicity studies and clinical trials all take time for a new compound and in the event of a novel virus or pandemic, weeks, and months matter. Furthermore, current lines of antiviral drugs including neuraminidase and polymerase inhibitors are associated with the emergence of antiviral resistance [22]. We need better therapeutic antiviral strategies for the effective management of viral diseases, while mitigating the emergence of drug resistance.

RNAi-targeting antiviral strategies can be developed as a first in class strategy as direct acting antivirals for RNA viruses. RNAi-based antivirals offer distinct advantages over traditional therapies due to their programmability, which allows rapid targeting of diverse viral RNA sequences within days. These properties position RNAi as a promising approach for combating viral infections in a timely manner. There is currently a dire need for the development of antiviral RNA therapeutics to control virus infections especially those with pandemic capacity, notably respiratory viruses. A modular therapeutic platform that can treat respiratory viruses, as well as their emerging variants could be a paradigm shift in the management of pandemics, but also in preparedness for future pandemics. Twenty months into the COVID-19 pandemic, we had only seen the first oral antiviral drug, molnupiravir, that shortens time to clearance of SARS-CoV-2 RNA and infectious virus in the nasopharynx and if administered early following infection, can reduce hospitalisation and death [23]. The first report of the activity of molnupiravir against SARS-CoV-2 was published in April 2021 demonstrating the long timeline required to advance from demonstration of efficacy in vitro and in animal models, to phase 3 efficacy studies. This is in vast contrast to the rapid testing of RNA-based vaccines for SARS-CoV-2 which only required the virus sequence and within 6 weeks, phase 1 studies for the Moderna vaccine [11] were underway. As with RNA vaccines, RNAi-based therapeutics can enter the clinical testing pipeline rapidly. This is evidenced by several siRNA drugs already in the market including the only oligonucleotide-based drug approved by the FDA in 2022 [24], AMVUTTRA™ (Alnylam Pharmaceuticals), an RNAi therapeutic for liver amyloidosis.

The greatest challenge is siRNA delivery. Therefore, there is an urgent need for new platforms that enhance RNA therapeutic efficacy, bioavailability and biodistribution that could be adapted rapidly to any emerging viruses. LNPs offer a great solution to this problem as it protects the siRNA cargo from degradation, bypassing the need to subject siRNAs to expensive chemical modifications. Indeed, we have taken this approach for siRNAs against SARS-CoV-2 and RSV [25–27]. Given the rapid advancements in RNAi technology and the increasing clinical success of such therapeutics, we anticipate a significant increase in RNAi-based treatments for viral diseases within the coming years. Last year, the FDA approved two antisense oligonucleotides (ASOs) for amyotrophic lateral sclerosis and polyneuropathy [6], marks significant milestones in nucleic acid based therapeutics, reinforcing the potential that RNAi may hold against as an agent against viral infections. Elebsiran (VIR-2218/ALN-HBV), an investigational siRNA drug targeting chronic hepatitis B (HBV) viral infection in the liver, is currently in Phase 2 clinical trials with excellent hepatic safety profile and reduction in HBV viral loads [28]. It is reasonable to design siRNAs that target chronic viral infections such as for HBV, to be heavily chemically modified to ensure higher retention time in target tissues to maximise its antiviral bioactivity. On the other hand, unmodified siRNAs may be ideal for targeting viruses with an acute infection sequalae (e.g., respiratory viral infections). In such situations, a cost-effective approach of encapsulating unmodified siRNAs in LNPs would be the best option. However, for chronic infections chemically modifying siRNA may be beneficial in allowing the siRNA to remain in higher bioavailability for longer. Another way of increasing siRNA antiviral activity and retention is by multiplexing siRNAs targeting multiple regions into one formulation as done previously against MARV [29, 30] and CHIKV [31]. This opens the possibility of also multiplexing multiple siRNAs targeting multiple viruses in a single LNP formulation. This exciting rational can be applied to respiratory viruses where co-infections in the respiratory tract does occur [32]. Nevertheless, the future of RNAi as a programmable antiviral therapeutic is the next frontier in RNA medicine. In the post-pandemic era, given the acceptance and uptake of mRNA vaccines, this will allow a more seamless transition of siRNA therapeutics in the clinic. Importantly, findings from the study with elebsiran [28] will open new avenues for other investigational antiviral siRNAs to enter this space. A recent review extensively delves into exploring siRNAs as an emerging class of antiviral drugs (see review by Chokwassanasakulkit et al., 2024 [33]). In comparison, our review focuses on the application of LNPs for the research and development as a suitable siRNA carrier tested in pre-clinical virus infection models.

### Lipid-nanoparticles (LNPs)

The fundamental knowledge of LNPs stem from the early discover of liposomes and lipoplexes, which support the fundamental roles that lipids have in the permeability of biological membranes [34]. As such, significant research has aimed to improve liposomes, with several being used in a number of clinical trials to deliver a variety of drugs [35, 36]. LNPs offer notable advantages over other delivery vehicles. Specifically, siRNA formulated in LNPs overcome issues associated with effective deliver of siRNA cargo, such as enhanced stability against nuclease degradation, targeted delivery to specific tissues, reduced immunogenicity, and favourable pharmacokinetics [37]. This platform avoids the risks of insertional mutagenesis associated with some gene therapies, such as the use of viral vectors as a delivery platform, and allows scalability for diverse applications, particularly against challenging viral targets. The application of such delivery platform to nucleic acids are versatile in nature allowing effective delivery of various types of RNA molecules. Importantly, the type of LNP choice may depend on the target tissue and to an extent the route of administration. Thus, the optimisation of LNPs can be complex, requiring a detailed understanding of LNP formulation chemistry and continuous refinement of the delivery system, especially for specific cell types or disease states [38]. However, this allows LNPs to be modular in nature whilst also allowing for up-scaled production with less variability.

Certainly, the scaling of LNP production has increased significantly over recent years and has established itself within the biopharmaceutical industry. Historically, LNPs have been produced using methods such as, bulk mixing, high-pressure homogenisation, high-speed stirring, microemulsion, solvent emulsification, and fluid-based methods [39]. Whilst instrumental in the advancement of LNP technology, they are associated with several limitations as they require multi-step procedures, resulting in high LNP variability and poor scalability, thereby hindering its clinical translatability [40]. The discovery of microfluidic methods of preparation are now commercially available, allowing for adaptable research within cell culture and pre-clinical models. This is advantageous in preserving expensive nucleic acids, both during synthesis and delivery, while maintaining characteristics that are shared within later stages of nucleic acid therapeutics (e.g., T-mixer). In microfluidic devices, lipids are dissolved in ethanol and nucleic acids in an aqueous buffer, which are then rapidly mixed through various mechanisms, such as sheath flow focusing, chaotic mixing, or integrated lipid nanoparticle preparation (iLiNP) devices. This allows for controlled ethanol dilution and uses lipid self-assembly mechanisms, thereby resulting in more uniform LNP characteristics, such as size, polydispersity index (PDI) and encapsulation efficiency. The type of LNP development approach may vary depending on context. For example, chaotic mixers and sheath flow devices may be more appropriate for initial research and development stages and pre-clinical screening, allowing for more precise control and release. Whereas in a T-mixer or iLiNP, are more suitable for bridging the gap to clinical translatability, yielding larger quantities of nucleic acids packaged into LNPs [41]. Cold chain storage and management also becomes paradigm, as differential conditions can influence preferential cell targeting or organ uptake, likely due to reorganization of the formulation [42]. The development of mRNA-LNP vaccines from Pfizer/BioNTech and Moderna each have different storage and transport requirements, which may suggest that specific formulations may alter or react differently, contributing towards fluctuations in LNP stability and effectiveness of nucleic acid delivery. Therefore, the mechanism of LNP self-assembly needs to be understood before it can be applied to downstream properties of LNP-based nucleic acid delivery. The surface charge of LNP is responsible for interaction of biological membranes. Generally, they are positively charged, although negatively charged LNPs also exist and have shown to accumulate in the spleen [43]. A known risk with LNP delivery platforms includes cytotoxicity and an unfavourable immune response, which can be caused by a highly positive surface charge or stimulation of the innate immune response [44, 45]. Table 1 summarises some key advantages and disadvantages of LNPs as a delivery platform.

**Table 1:** Advantages and disadvantages of LNPs as a therapeutic delivery system

Advantages	Disadvantages
Enhanced stability by protecting against host-mediated enzymatic degradation [37].	Requires understanding of formulation science due to complexity of self-assembly, whereby diverse lipid chemistries and nucleic acid properties can complicate characterization and optimisation
LNPs enable efficient encapsulation and targeted delivery to the intended site of action.	Storage and stability [42]
LNPs offer modularity, allowing for the incorporation of several small molecules such as siRNA, diversifying and enhancing their versatility given experimental context.	LNPs may still face challenges in achieving effective gene silencing in specific cell types or disease states [38]
Several nucleic acid therapeutics packaged into LNPs are already in the clinic or currently in active clinical trials [46]	Risk of cytotoxicity or unwanted immune responses, especially with certain LNP formulations [44, 45]
Advances in methods of preparation contributes towards a scalable approach of nucleic acid delivery [47, 48, 49]	

### LNP - the basics

**Fig. 1:**
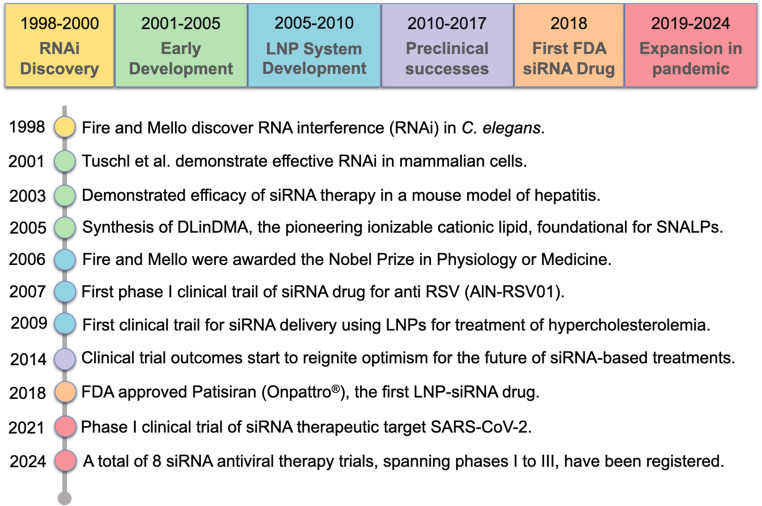
Structure and Functional Mechanisms of LNPs in siRNA Delivery. The upper panel provides an overview of LNP constituents, including ionizable lipids, helper lipids, cholesterol and PEG lipids, each contributing towards the structural and functional properties of the LNP. The core contains the nucleic acid cargo (siRNA), which is encapsulated for delivery. The right panel explores the role of PEG lipids with variations in alkyl chain length (e.g. DSPE-PEG2000 vs. DMG-PEG2000) modulating desorption kinetics. The central diagram outlines the important role of ionizable lipids in siRNA delivery. The bottom panel highlights site-specific delivery through varying targeting ligands or moieties, such as GalNAc for the delivery to liver hepatocytes

LNPs are made up of four lipid groups: ionizable lipids, helper lipids, cholesterol, and PEG lipids. Figure 1 outlines the basic constituents of LNPs, with examples focussing on the roles that ionizable lipids and PEG lipids play in LNP functionality. Briefly, the particle size of LNPs is between 10 and 400 nm, or 10 to 150 nm for systemic delivery via intravenous (i.v) injection. Smaller LNPs are more prone to aggregate, but the larger surface-to-volume ratio promotes faster drug release. Particle surface charge influences the interaction between the LNP and cellular membranes and is defined by the value of its zeta-potential [35]. Unlike liposomes, LNPs utilise ionizable lipids which are crucial for the encapsulation and delivery of nucleic acids. Lipoplexes on the other hand are lipid-nucleic acid complexes that are formed through electrostatic interaction, often resulting in less structural stability and has issues in translation to pre-clinical models [50]. LNPs build upon liposome technology by incorporating the following components which are discussed below. Unlike lipoplexes, LNPs have the capacity to transition from a neutral to cationic charge in a pH dependant manner, therefore giving more controlled release of the nucleic acids.

#### Ionizable lipids

Ionizable cationic lipids are important in facilitating nucleic acid encapsulation and endosomal membrane disruption, facilitating cytosolic release of nucleic acids. The mechanism of endosomal escape has not been completely understood, but the idea of cationic lipids promoting the fusion process with an anionic membrane is what leads to effective cargo release. Ionizable lipids bear a tertiary amine that is not-charged under neutral conditions and positively charged in pH levels that is below its acid-dissociation constant (Ka) of the lipid, giving the LNP its cationic properties [51]. Therefore, its efficiency depends on a pH-responsive behaviour and is considered one of the most influential factors in facilitating gene silencing [52]. Early discovery and synthesis of ionizable lipids, DODAP, were not feasible for the delivery of nucleic acids, due to the high dose required. In the development of ionizable lipids, 1,2-dilinoleyloxy-N, N-dimethyl-3-aminopropane (DLinDMA) and its ester-containing analog, 1,2-bis(linoleoyloxy)-3-(dimethylamino)propane (DLinDAP) were important in understanding the structure-function relationship that governs nucleic acid delivery efficiency [53]. The incorporation of ester bonds in the hydrophobic tail would offer theoretical advantages, such as rendering the lipid more susceptible to hydrolytic cleavage under physiological conditions. However, the practical drawbacks, as demonstrated in pre-clinical studies, included reduced delivery efficacy and compromise to the lipid structure [51]. This underscores the importance in lipid stability to achieve reliable gene-silencing, leading to the development of subsequent lipids such as (6Z,9Z,28Z,31Z)-Heptatriaconta-6,9,28,31-tetraen-19-yl 4-(dimethylamino) butanoate (DLin-MC3-DMA), with a pKa 6.44, the same formulation that is used in Onpattro^®^ [54]. This study proved that the optimal siRNA encapsulation and release was dependant on having an optimal pKa value of 6.2–6.5. The pKa of ionizable lipids can be fine-tuned through chemical modifications or other LNP constituents such as zwitterionic helper lipids and cholesterol.

#### Helper lipids

Helper lipids are a collective group, that encompasses sterols, phospholipids and glycerolipids. Much like the other LNP constituents, the roles of helper lipids have currently been eluded towards providing stability during storage and circulation. One example of a commonly used helper lipid is 1,2-dioleoyl-*sn*-glycero-3-phosphoethanolamine (DOPE), which has shown to promote endosomal release of oligonucleotides [55]. Phospholipids on the other hand contribute towards the formation of a stable lipid bilayer structure in LNPs [51].

#### Cholesterol

Cholesterol is a naturally occurring sterol that is also used in LNP formulations. The role that cholesterol can play largely varies depending on the therapeutic application. However, its overarching function is to pull the lipids towards a liquid-ordered phase. This is a phase that is characterized by decreased membrane fluidity and increased bilayer thickness [56]. As cholesterol increases membrane rigidity, it serves as a mechanism to reduce leakage from the liposomal core, thereby resulting in better circulation, half-life, and encapsulation efficiency [12, 57]. Originally it was thought that a certain concentration of cholesterol (40 mol%) was needed to achieve encapsulation efficiency, although recent studies have determined that nucleic acid encapsulation may occur in lower concentrations than that. Rather, the findings suggests that if the concentration of cholesterol exceeds its capacity for stable incorporation into the LNP membrane, insoluble cholesterol crystals may form within the LNP core, paradoxically compromising its structural integrity [58, 59].

### PEG lipids

Polyethylene glycol (PEG)-lipids are also commonly used in LNP formulations, constituting only a small molar percentage of the final LNP types, although influences several key properties including, population size, dispersity, LNP aggregation and overall stability during preparation and storage [12]. The conjugation with PEG has also shown to enhance pharmacokinetic profiles and significantly slower renal clearance as compared to naked siRNA [60]. LNPs are formed by electrostatic association between the negatively charged nucleic acids and the positively charged lipids. These then strengthen through hydrophobic interaction and van der Waals forces between the lipid components. For example, the PEG-lipid component can interact with the hydrophobic tail, a longer length of these alkyl or acyl chains influences stability [12]. Some common issues encountered with an LNP delivery platform may arise due to poor endosomal escape into the cytosol before degradation or non-specific cellular uptake. The process of PEGylation has been shown to inhibit cellular uptake and endosomal escape, thus may be considered a double-edge sword [61]. Certain strategies may be utilised to circumnavigate these issues by fine-tuning the PEGylation process, such as shortening of the alkyl chain [62–64]. A shorter alkyl chain, such as 1,2-dimyristoyl-rac-glycero-3-methoxypolyethylene glycol-2000 (DMG-PEG_2000_), leads to faster desorption kinetics and quicker siRNA release [64]. Whereas a longer alkyl chain will have an extended half-life, such as 1,2-distearoyl-sn-glycero-3-phosphoethanolamine-N-[methoxy(polyethylene glycerol)-2000] (DSPE-PEG2000), as it will prolong its bioavailability by remaining in circulation for longer. This prolonged circulation can increase the chance of LNPs reaching their target cells, however, may also increase the risk of cytotoxicity due to accumulation in non-target tissues. Despite this, it still appears that correctly PEGylating an LNP does not seem to affect gene editing, as previously demonstrated when combined with N-acetyl-D-galactosamine (GalNAc), a common LNP modification used for liver-targeting [65]. Indicating that specific tissue targeting is more dependent on the overall formulation rather than the length of the alkyl chains.

#### Interplay between LNP constituents

Early works propose the molecular shape hypothesis, which suggests that micellular-like packing of ionizable lipids around the nucleic acids, exhibiting a cone-like geometry of their self-assembled structures [66]. However, the true structure and arrangement of the lipids are not fully known. Several methods have been used to investigate LNP structure and lipid arrangement, such as, cryogenic electron microscopy (Cryo-TEM), molecular dynamics (MD), small-angle neutron scattering (SANS), small-angle x-ray scattering and nuclear magnetic resonance spectroscopy (NMR) (see review by Albertsen et al. 2022 [12]). Ionizable lipids form the core with the siRNA, whilst the helper lipids, cholesterol and PEG-lipids contribute to the outer shell. The choice of helper lipids will significantly influence stability and fusion capability of LNPs, with small changes drastically changing function. For example, cone-shaped lipids like DOPE promote endosomal release, whereas cylindrical lipids like DSPC enhance bilayer stability [12, 55]. DOPE-containing lipids LNPs have stronger interactions with apolipoprotein E (ApoE), thus will accumulate in the liver as ApoE facilitates LNP uptake through the low-density lipoprotein receptor (LDLR), compared to DSPC which targets the LNPs to the spleen. A study using identical ionizable lipids, but varying helper lipids alter overall biodistribution and efficacy, thereby demonstrating the role helper lipids may have in facilitating ionizable lipids for endosome escape [67]. Contrastingly, a recent study showed that variation in PEG-lipids will significantly influence LNP characteristics, overshadowing the effects of helper lipids. Increasing the PEG-lipid content from 2 to 10% creates a smaller particle size, higher PDI, lower encapsulation efficiency and reduced shelf-life [68]. Therefore, the specific formulations of certain constituents, such as helper lipids are likely functionally relevant in how they interact with PEG-lipids. For example, a higher PEG-lipid content can disrupt the ordered multilamellar structure that is formed by helper lipids, leading to less ordered and smaller nanoparticles.

### LNPs in the delivery of siRNA

*Ionizable LNPs – An ideal platform for siRNA delivery.*

Delivery of siRNA encounters several problems due to challenges associated with bioavailability at the target site. siRNA is small in size (13 kDa), making it susceptible to renal clearance. They are also a negatively charge molecule, thus it is difficult for siRNAs to cross biological barriers, into the cytoplasm where siRNA is therapeutically functional [69].

Certain subsets of cationic lipids are efficient at specific targeting of hepatic cells, specifically the use of LNPs that contain the cationic aminolipid XL-10 which has been used in several siRNA-LNP formulations for liver targeted delivery [47, 70, 71]. Similarly, LNP modifications that target hepatocyte-specific ligands, GalNAc, have a high affinity for the asialogycoprotein receptor (ASGPR) that is predominantly expressed in hepatocytes, making them ideal for targeting the liver for disease or viral infection, such as hepatitis viruses which predominantly infects the liver [72]. Parallel to liver targeting, ionizable cationic lipids have been engineered for siRNA delivery to the lungs, offering new avenues for treating lung diseases. For example, DOTAP with the ionizable lipid DLin-MC3-DMA (dmLNP) based LNPs can efficiently encapsulate and deliver siRNA to lung epithelial cells, offering a targeted approach for treating respiratory viral infections [73]. The use of DOTAP is commonly used for localised delivery, as DOTAP being permanently cationic, may interact with negatively charged blood components when delivered systemically, thereby leading to ineffective delivery.

Stable nucleic acid-lipid particles (SNALPs) have been used as a means for encapsulating siRNA, through the process of spontaneous vesicle formations via the hydrophobic and hydrophilic properties of the lipid molecules [51, 74]. Typically having a mean size of 100 nm, a neutral charge and made with an ionizable cationic lipid, helper lipid and PEG-derived lipid. These are characterised as having high encapsulation efficiency, but also may be engineered to promote targeted delivery. One advantage of SNALPs is the simplicity associated with their capacity for self-assembly, this detracts from the specificity that may be needed in other therapeutic contexts, such as the incorporation of ligands for cell-specific ligands. SNALPs are predominantly build on ionizable cationic lipids. In contrast, polyplexes leverage cationic polymers such as Polyethyleneimine (PEI) to condense RNA or DNA content into nanoparticles known as polyplexes [75, 76]. PEI is not a traditional component of LNPs, rather used in hybrid lipid nanoparticles or lipid-polymer hybrid nanoparticles (LPHNPs). PEIs contain protonable groups, which causes destabilization of the endocytic membrane, thereby facilitating transport from the late endosome into the cytosol. Such hybrid nanoparticles integrate characteristics of both, utilising lipids for enhanced delivery and polymers for structural stability. PEI based LNPs may also be combined with PEGylating constructs to further improve delivery efficiency [77].

Another novel LNP is the lymphocyte membrane-and 12p1-dual functionalized siRNA delivery lipid nanoparticle system (MPLN). These are derived from components of the lymphocyte cell membrane, taking advantage of the natural homing properties lymphocytes possess to specifically target tissue and evade the immune system [78]. The peptide 12p1 is combined with a T cell membrane, which shows competitive binding with gp120, a human immunodeficiency virus (HIV)-1 glycoprotein [78, 79]. These type of LNPs could serve as a new class of naturally derived biocompatible LNPs for therapeutic nucleic acid delivery. A major limitation of using lymphocyte membranes, is the complications with large-scale manufacturing and quality control. Outside of HIV targeting, this system may fail to exhibit the same targeting efficiency, thereby hindering their broader applicability. Stearylamine/octadecylamine (ODA) is another cationic lipid formulation that was recently tested for siRNA delivery [80]. As cationic lipids, these lipids are categorized based on the nature and charged density of hydrophilic head groups. ODA has a cationic charge in LNP for better encapsulation siRNA. However, due to their high positive charge density, there may be cytotoxic build-up, immunogenicity and rapid clearance hereby leading to poorer bioavailability. Furthermore, ODA formulations may not be optimisable for tissue-specific targeting.

### Administration routes of LNPs

The route of administration is important to consider when delivering RNA-loaded LNPs. As such, various routes of administration may be exploited to achieve site-specific delivery of LNPs. These are categorised as either a local or systemic route of administration. These routes of delivery are determined by the formulation properties and therapeutic indicators. If the LNP size is greater than 100 nm, they are taken up by the reticuloendothelial system (RES), thereby accumulating in the lung, liver, spleen and bone marrow. Whereas smaller LNPs will be in circulation for longer [81].

Systemic administration can be used to prime a systemic immune response, such as intradermal (i.d.), intramuscular (i.m.) and subcutaneous (s.c.), causing resident and recruited antigen-presenting cells (APC) hence are used for vaccinations to delivery mRNA-encoding antigens. Vascular and lymphatic vessels in the tissue help the APCs and mRNA circulate towards the lymphatic vessels, to stimulate T cell immunity [82]. The caveat is whilst this provides a robust immune response, cytotoxicity may become an issue [45]. Other forms of nucleic acid therapies are outside the scope of this review, however a recent study demonstrated the development of anti-PEG antibodies in response to mRNA vaccines [83]. Whilst it did not impact the overall effectiveness of the immune response, development of anti-PEG overtime could paradoxically lead to quicker clearance and an unfavourable immune response. Hence, something which may be considered when formulating LNPs with PEG lipids.

Generally, systemic delivery of siRNA may require a much higher dose would be needed to preserve bioavailability by protecting the therapeutic payload from endonuclease-mediated degradation, off-target endocytosis and escape or unwanted immune stimulation, in turn-causing a tighter therapeutic window. Despite this, Patisiran (ONPATTRO™) delivers siRNA targeting transthyretin (TTR), for the treatment of TTR-type familial amyloid polyneuropathy. This was achieved through systemic LNP-siRNA delivery via intravenous injection (i.v) supressing the TTR gene responsible for deposition of amyloid fibrils and misfolded TTR [84]. As a result, systemic delivery is used in the context of viral infections that impact internal organs, such as the liver, lungs and spleen. For example, adenoviruses [71] and hepatitis B [85–88] viral therapies may benefit from the natural tendency of LNPs to accumulate within the liver, where viral replication dominantly occurs. Similarly, for viral infections that have high viral loads or can cause severe infection. Post-exposure treatment models of viral infections, such as Ebola [89–92] and Marburg [29, 30, 93] virus, aim to maximise bioavailability and rapid onset of therapeutic infection. Local administration of siRNA can be used to treat diseases or viral infections that require organ specific delivery, minimizing unwanted immune responses, whilst also enhancing therapeutic efficacy. For example, intranasal delivery of LNPs, may harness mucosal immunity for vaccine development, but also for delivery of siRNA targeted towards the nasal and lung region. For respiratory viral infection models, the viral load will be highest in the upper and lower respiratory tract. Delivery of siRNA-LNP via the i.n route has demonstrated strong antiviral effect in the nasal and lung cavity [26, 27]. Although, in clinical end stage, i.v delivery could also be used to maximise clinical rescue in severe respiratory viral infection [25].

### The current landscape of preclinically tested siRNA-LNP antiviral therapies

**Fig. 2:**
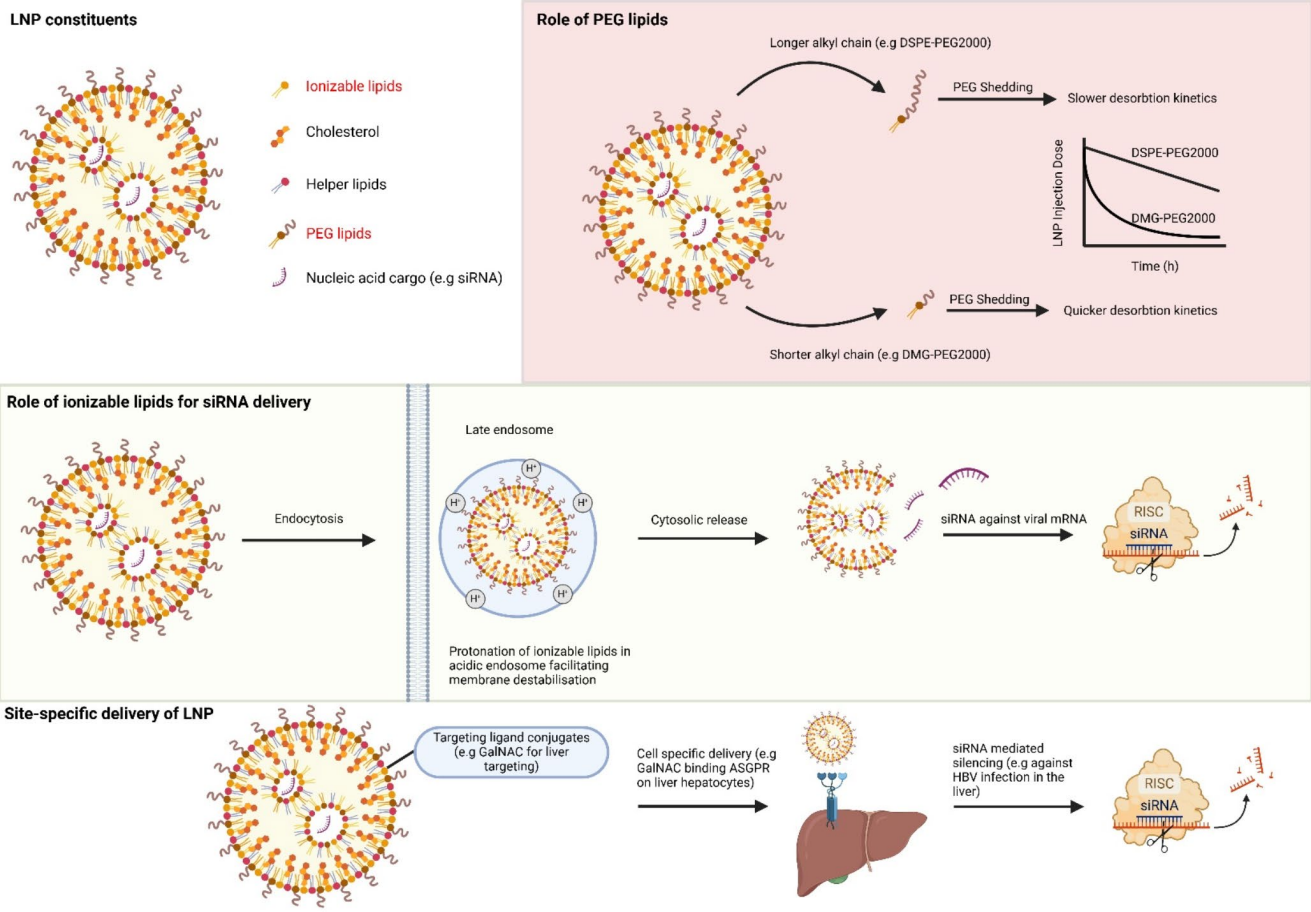
Timeline summarizing different stages in the course of development of SiRNA-LNP therapeutics against viruses. FDA, Food and Drug Administration; RNAi, RNA interference; siRNA, small interfering RNA; LNP, lipid nanoparticle; DLinDMA, 1,2-dilinoleyloxy-N, N-dimethyl-3-aminopropane; SNALPs, Stable nucleic acid-lipid particles; RSV, respiratory syncytial virus

The development of siRNA-LNP therapies for treating viral diseases has seen significant milestones, transforming the landscape of antiviral treatment (Fig. 2). Key events include the successful demonstrable ability for siRNAs to silence virus-specific genes in early laboratory studies [1, 94–96], followed by the optimization of stable LNP carriers to improve the delivery and effectiveness of siRNAs [97, 98]. A limited number of human clinical trials have further established the safety and potential effectiveness of antiviral siRNA therapies in reducing viral loads (see review by Kang et al., [99]) albeit with varying successes.

**Fig. 3:**
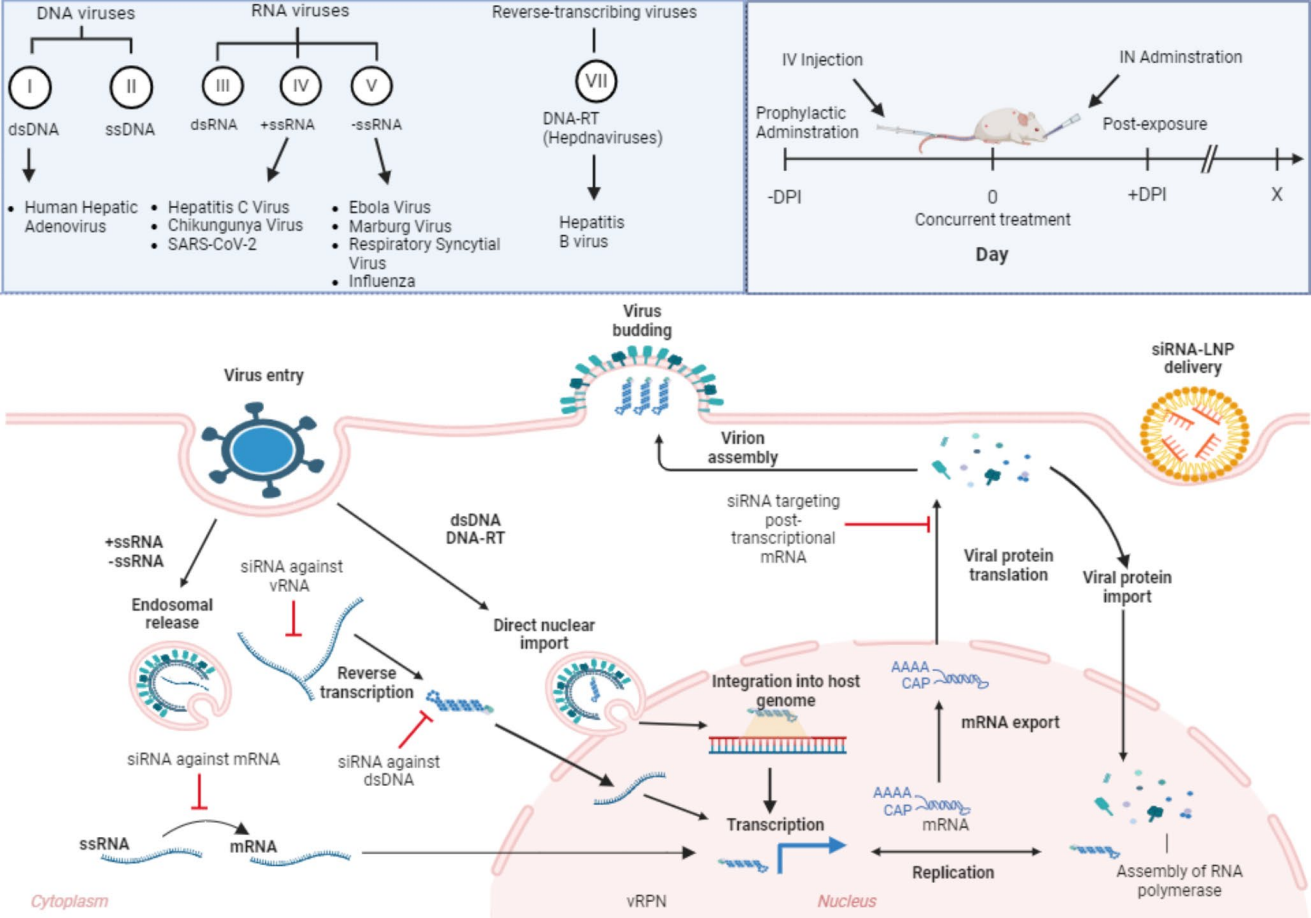
Strategic Intervention of siRNA Therapeutics Across Viral Life Cycles and Delivery Timelines. The upper panel classifies viruses by genetic content and replication strategy according to the Baltimore classification, with disease examples for each category. The central diagram delineates the siRNA mechanism of action during viral infection, including entry, replication, and exit, while highlighting potential siRNA intervention sites. The top right panel depicts routes of siRNA-LNP administration include intravenous (IV) directly into the bloodstream, intraperitoneal (IP) into the peritoneal cavity, subcutaneous (SC) into subdermal fat, and intranasal (IN) through nasal delivery. The timeline illustrates optimal windows for prophylactic and post-exposure siRNA-LNP treatment, underlining the versatility of siRNA therapeutics in experimental and clinical settings

Transitioning to the current scope of research, the use of LNPs and siRNAs in non-human primate models has expanded our understanding of combating viral infections at the genetic level. This approach provides key advantages, given the diverse mechanisms and life cycle of viruses, that otherwise pose challenges to traditional antiviral strategies. In summary, siRNA-LNP therapies offer a unique and powerful means to interfere with viral replication and dissemination at various stages (Fig. 3), underlining their versatility and precision as essential components in our fight against viral diseases. The array of administration routes and strategic timing of interventions further highlights the adaptability and significance of siRNA-LNP therapies in preventive and therapeutic settings, marking them as a forefront tool in the management of viral diseases.

In a rapidly evolving field of antiviral research and development, LNP mediated delivery of antiviral siRNA represents a cutting-edge approach for the treatment of viral infections. Table 2 provides an overview of current in vivo studies that have been conducted to date, showcasing viral target diversity, efficacy of siRNA-LNP formulations and their clinical outcome.

**Table 2:** Preclinical testing of antiviral siRNA-LNPs in animal models

Modification	Virus model	Viral target(s)	Treatment regimen	LNP characteristic	Outcome	Reference
2′-O-methyl, phosphorothioate (PS)	Adenovirus (Ad5) in Syrian hamsters	hAd5 pTP (sipTPmod)	Co-treatment, 2 mg/kg i.v.	PEG LNP with XL-10, Size: 83.2 nm, PDI: 0.04, Zeta: 0.7 mV, EE: 90%	Decreased viremia, Reduced liver inflammation	Geisler et al. [71]
Unmodified and 2′-O-methyl	Ebola (ZEBOV) in Rhesus macaques	Polymerase L, VP24, VP35	Post-exposure, 2 mg/kg i.v.	SNALP with DSPC, PEGC-DMA, DLinDMA	Improved survival, Symptom reduction	Geisbert et al. [90]
Unmodified	Ebola (ZEBOV) in Guinea pigs	L Gene	Post-exposure, 1 mg/kg i.p.; Prophylactic, 8 mg/kg i.v.	SNALP, Size: 81 nm, PDI: 0.11, EE: 92%	Ablated plasma viremia, Enhanced survival	Geisbert et al. [89]
Unmodified	Ebola (Makona) in Rhesus macaques	VP35, Lpol	Post-exposure, 0.5 mg/kg i.v.	LNP with PEG-C-DMA, DLin-MP-DMA	Increased survival from lethal challenge with mild symptoms, Reduced plasma viremia	Thi et al. [92]
Unmodified	Sudan Ebola (SUDV) in Rhesus macaques	VP35, NP, VP24	Post-exposure, 0.5 mg/kg i.v.	LNP with PEG-C-DMA, DLin-MP-DMA	Superior protection and symptom amelioration, increased survival from lethal challenge	Thi et al. [91]
2′-O-methyl	Marburg virus (MARV) in Rhesus macaques	NP gene	Post-exposure, 0.5 mg/kg i.v.	SNALP, Size: 78–79 nm, PDI: 0.04–0.09, EE: 97–98%	100% survival, Reduced plasma viremia	Thi et al. [29]
2′-O-methyl	Marburg virus (MARV) in Guinea pigs	NP, Lpol, VP24, VP40	Post-exposure, 0.5 mg/kg i.v.	SNALP, Size: 78–79 nm, PDI: 0.04–0.09, EE: 97–98%	60-100% survival, Reduced plasma viremia	Ursic-Bedoya et al. [30]
2′-O-methyl	MARV and RAVV in Rhesus Macaques	MARV and RAVV NP protein	Post-exposure, 0.5 mg/kg i.v.	SNALP, Size: 78–79 nm, PDI: 0.04–0.09, EE: 97–98%	100% survival, 4 log reduction in viremia, less severe symptoms	Thi et al. [93]
Unmodified	Chikungunya virus (CHIKV) in mice	E2, NS1 genes	Post-exposure, 1 mg/kg i.v.	Commercially available HiPerFect Transfection Reagent (QIAGEN)	Significantly reduced CHIKV titer in serum and muscle tissues, preserved the morphological integrity of the muscle tissues with regeneration	Parashar et al. [80]
Unmodified	Chikungunya virus (CHIKV) in mice	E2, NS1 genes	Post-exposure, 1 mg/kg i.v.	Various PLGA and lipid formulations	Reduced inflammation, necrosis, 99% viremia reduction	Jeengar et al. [31]
2’-O-methyl	Chimeric mouse model of Hepatitis B (HBV)	ARB-1740 - HBV surface antigen (HBsAg), hepatitis Be antigen (HBeAg) and HBV core-related antigen (HBcrAg)	Post-exposure, 0.3 mg/kg − 3 mg/kg, i.v.	1,2-distearoyl-sn-glycero-3-phosphocholine, poly(oxy-1,2-ethanediyl), α([2,3-bis(tetradecyloxy)propyl]amino carbonyl)-ω-methoxy, 3-dilinoleylmethoxy-N, N-dimethylpropan-1-amine, and cholesterol Mean particle size = 65–80 nm Polydispersity = < 0.1 siRNA encapsulation efficiency = 92–98%	Reduction in serum HBV DNA, HBsAg, serum HBeAg, and liver total HBV RNA.	Thi et al., [88]
Unmodified	HBV mouse model	Genotypes A, B, C regions	Post-exposure, 5 mg/kg, i.v.	N-acetyl-d-galactosamine (GalNAc)/PEG-LNP	Reduced serum HBsAg, HBeAg, HBcrAg levels	Sato et al. [85]
2’-O-methyl	Hep-B/Hep-D in chimeric mouse models	ARB-1740 siRNA against HBV/HDV	Post-exposure: 3 mg/kg, i.v.	LNP, Size: 65 to 80 nm, PDI: <0.1, EE: 92 to 98%	Reduced HBV viremia and serum HBsAg, Decreased HDV viremia	Ye et al. [86]
2’-O-methyl, 2’-Fluoro	HBV in multiple mouse models	siRNA targeting multiple transcripts of HBV genes, called SR16-X2M2 or siHBV	Post exposure: multiple doses via i.v.	RBP131 – LC8, PEG2000 PE, cholesterol, Size: 60–100, PDI: < 0.20, EE: > 90%, Zeta: 0.089 ± 0.342	Levels of blood and liver S antigen of HBV (HbsAg) were significantly reduced	Huang et al. [87]
Unmodified	Human and avian influenza viruses in mice	Influenza nucleoprotein (NP) and PB1 genes	Co-treatment, virus was combined with oligofectamine/siRNA (final 1.51 nmol siRNA), i.n.	Oligofectamine (commercially available)	Significantly reduced lung virus titres in infected mice and protected animals from lethal challenge	Tompkins et al. [100]
Unmodified	SARS-CoV-2 in mice	Highly conserved regions of SARS-CoV-2 (RdRp, helicase region and 5’ UTR)	Post-exposure 1 mg/kg i.v.	DOTAP with the ionizable lipid DLin-MC3-DMA (dmLNP) DOTAP, cholesterol, DOPE, and PEG2000-C16 Ceramide (stealth LNPs (sLNPs))	Repression of virus replication in lungs, pronounced survival advantage	Idris et al. [25]
Unmodified	SARS-CoV-2 and RSV in mice	Highly conserved regions of SARS-CoV-2 (RdRp, helicase region and 3’ UTR) and RSV P gene	Prophylactic, 1 mg/kg, i.n and i.v.	DOTAP with the ionizable lipid DLin-MC3-DMA (dmLNP) DOTAP, cholesterol, DOPE, and PEG2000-C16 Ceramide (stealth LNPs (sLNPs))	Significant reduction in lung RSV and SARS-CoV-2 viral loads with siRNA delivered by i.n.	Supramaniam et al. [27]
Unmodified	SARS-CoV-2 in mice	Highly conserved regions of SARS-CoV-2 (helicase region)	Prophylactic, 1 mg/kg, i.v and i.n	DOTAP with the ionizable lipid DLin-MC3-DMA (dmLNP)	Significant reduction in lung viremia and reduction in viral loads in the nasal cavity with i.n. administration of siRNA	Idris et al. [26]

#### Human adenovirus

Human adenovirus (hAdv) belongs to the *Adenoviridae* family (Mastadenovirus genus) and is a dsDNA virus (Class I). It can infect several organ types, due to its wide tissue tropism. The common sites of infection are the respiratory tract, corneal epithelia, and the intestinal tract. hAdv internalizes by binding receptor-mediated endocytosis before releasing the virion through endosomal escape within the cytoplasm where it is then directed into the nucleus via nuclear localization signals, causing viral genomic release. Infection with hAdv is generally treated symptomatically as infections are self-limiting. However, hAdv can pose a risk for the immunocompromised, elderly, and younger children, leading towards a more serious disease state in these at-risk groups [101]. Infection with hAdv has also been implicated in the development of various clinical syndromes, including the development of community acquired pneumonia. As infection with hAdv has been observed in viral co-infections, thereby augmenting severity [102]. There are currently no approved treatments, with limited efficacy shown in off-label treatments such as Ribavirin, Cidofir and Ganciclovir (see review by Dodge et al., [103]). Using siRNAs directed against hAdv precursor terminal protein (pTP) or polymerase (*pol*) gene and packaged in PEG-LNPs, siRNA-LNP treatment in a type 5 hAdv (hAd5) infection model in Syrian hamsters efficiently inhibited viral replication in the liver and reduced hepatic inflammation [71]. The anti-hAdv siRNA used was chemically modified with 2-O-methyl modifications as well as phosphorothioate (PS) linkages and the observed antiviral efficacy (serum and liver viremia) was irrespective of the hAdv challenge dose (low vs. high inoculation dose). This underscores the potency of this siRNA in a post-infection therapeutic intervention. Moreover, it demonstrates the diverse application of siRNA as an antiviral agent against other viruses with lesser focuses on antiviral development.

#### Filoviruses - Ebola virus, Marbug virus and Ravn virus

Ebola virus (EBOV) is a single-stranded negative sense RNA virus (Class V), thus must transcribe their RNA into complementary positive-sense mRNA before translation. EBOV belong to the Filoviridae family and is commonly found in the Sub-Saharan African region (case fatality rate ~ 70%). Alarmingly, individuals with respiratory, neurological, or haemorrhagic symptoms have a higher risk of death [104]. To date, there are no approved vaccine or therapeutic treatment modalities available for preventing or managing EBOV infections. EBOV genomic contents consist of the nucleoprotein (NP), VP35, VP40, glycoprotein, VP30, VP24 and the L protein. The L and VP35 proteins are involved in the formation of the polymerase complex, thus has been a long-standing target for RNAi therapeutics against EBOV. Single siRNAs against VP24, L polymerase and VP35 as well as a combined siRNA cocktail against one or more of these targets have been shown to survivability and reduced symptomology in non-human primate models when given across varying post-exposure treatment regimens and significantly reduced blood plasma viremia [89–92]. The EBOV non-human primate (NHPs) model is a lethal challenge model. Indeed, these anti-EBOV siRNAs as a single formulation (largely unmodified), packaged in either SNALPs or PEG-LNPs, were able to protect NHPs against the lethal EBOV challenge when administered post-exposure. However, this does not translate well in patient human trials [82], especially for EBOV patients in the late stages of the disease where blood viremia levels are high. Perhaps such interventions may be beneficial during the early stages of the disease. However, the formulation of LNPs used prioritises higher circulation and half-life, ensuring robust delivery and prolonged systemic availability.

Like Ebolavirus, Marburg virus (MARV) and Ravn virus (RAVN) are also filoviruses and negative-sense single-stranded RNA viruses. MARV also causes severe haemorrhagic fever and severe disease in humans, carrying a high mortality rate [105]. MARV has an almost similar genomic structure as EBOV. Post-exposure using a MARV NP gene targeting siRNA, a 2’-O-methyl modified siRNA packaged into SNALPs consisting of cationic lipids (1,2-dilinoleyloxy-3-N, N-dimethylaminopropane), helper lipids (Dipalmitoylphosphatidylcholine) and PEGylated lipids (3-N-[(ω-methoxy poly(ethylene glycol)2000)carbamoyl]-1,2-dimyrestyloxy-propylamine), resulted in a 100% survival rate in NHPs challenged with MARV [29, 90]. This LNP formulation has been consistently used displaying high potency in targeting filovirus-infected NHPs. Similarly, an siRNA cocktail of two independent anti-MARV targeting siRNAs targeting the NP gene also provided better clinical survival and protection compared to NHPs treated with single siRNA formulations [30]. Indeed, administration of a single siRNA-LNP formulation was sufficient to completely rescue RAVN infected NHPs from lethality (100%) [93], highlighting the potency of this siRNA in vivo. These LNPs featured ionizable lipids for pH-responsive delivery and PEGylated lipids for extended circulation stability.

#### Chikungunya virus

Chikungunya virus (CHIKV), an alphavirus, is a positive-sense single-stranded RNA virus (Class IV), that uses its own viral RNA as mRNA to directly express viral protein. Its genome consists of non-structural proteins (nsP1, nsP2, nsP3 and nsP4), needed for viral replication; structural proteins (E1, E2, E3 and 6 K), required for capsid and envelope protein formation. CHIKV is a mosquito-borne virus that causes Chikungunya fever (headache, joint pain, rash, and arthralgia). E2 and ns1 were identified as being highly conserved across different CHIKV strains and siRNAs complexed in HiPerFect, a commercially available transfection reagent containing a blend of cationic and neutral lipids, administered in CHIKV infected mice 3 days post-infection resulted in significant reduction in viral loads in serum and tissue muscles [80]. These same siRNAs were then later packaged into in-house generated cationic LNP formulation, ODA. Post-exposure delivery of these siRNAs through the IV route significantly reduced plasma viremia, where the combination of both siRNAs (targeting E2 and NS1) as a cocktail formulation completely inhibited viral replication [31].

#### Hepatitis B

Hepatitis B (HBV), a double-stranded RNA virus (Class VII), that uses a covalently closed circular DNA (cccDNA) intermediate. The formation of this cccDNA contributes towards the persistence and treatment resistance of HBV as it allows for the virus to enter a stable and protected state. Presently, there are direct-acting antiviral agents (DAAs) and nucleotide/nucleoside analogues (NUCs) being used for the management of HBV and HCV (see review by Schlaak et al., [106]). Although, these face their own challenges, such as incomplete viral eradication and resistance, as small-molecule therapies targeting proteins may generate resistant mutants. Whilst these drugs are effective at inhibiting viral replication, they do not appear to be effective at completing eliminating HBV infection due to the formation of its cccDNA. It is the cccDNA form of HBV that serves as a template for subsequent rounds of replication and more importantly the production of HBV surface antigen (HBsAg) [107]. Whilst these antigens are non-infectious, the secretion of such antigens are important in host immunosuppression, as a result allowing HBV to establish and maintain chronic infection. ARB-1740 is a preclinical stage pan-HBV targeting siRNA agent that includes a cocktail of three siRNAs packaged in LNPs, targeting HBV surface antigen (HBsAg), hepatitis Be antigen (HBeAg) and HBV core-related antigen (HBcrAg) [88]. Importantly, ARB-1740 has pan-activity across all HBV genotypes A–H. In a human chimeric PXB mice infected with HBV genotype C, administration of ARB-1740 resulted in the significant reduction in serum HBV DNA and HBsAg, serum HBeAg, and liver total HBV RNA. In another study, a single dose cocktail targeting against the conserved regions against genotypes A, B and C using this siRNA led to a significant decrease in expression of those antigens [85]. Similarly, using the same siRNAs packaged in LNP modified with a hepatocyte-specific ligand, GalNAc, lowered viremia in serum and HBsAg expression in Hepatitis B and Hepatitis B delta (HDV) co-infected humanized mice models [86]. The formulation of this LNP notably included YSK13-C3, a pH-sensitive ionizable lipid, DMG-mPEG2000 and DSG-PEG2k-GalNAc3. The conjugation with GalNAc ensures targeted interaction with the ASGPR, critical for effective liver targeting. A study by Huang et al., showed that siRNA targeting multiple transcripts of HBV genes (SR16-X2M2) reduced levels of blood and liver HBsAg in multiple HBV animal models [87]. The LNP was developed using a novel ionizable lipid-like material (lipidoid), LC8, for delivery to hepatocytes. LC8, was formulated with DPPE-mPEG2000 (16:0 PEG2000 PE), and cholesterol can form LNPs, termed RBP131 in that study. Though the human clinical trials for JNJ-73,763,989 (JNJ-3989), an siRNA that targets all HBV RNAs failed to seroclear HBsAg, most tested HBV patients treated with JNJ-3989 had demonstrable reductions in HBsAg that can potentially improve the liver environment conducive to better immune control [108].

#### Influenza virus

Influenza also transcribes its negative-sense single-stranded RNA into positive-sense mRNA (Class V). There are currently four classes of approved antivirals used for the treatment of influenza, these include: adamantanes, neuraminidase inhibitors, RNA dependent polymerase (RdRP) inhibitors and polymerase acidic endonuclease inhibitors [109]. Due to increasing antiviral resistance and waning efficacy of these existing antivirals due to rapidly mutating nature of influenza viruses, the development in better direct-acting anti-influenza therapies are warranted. For both influenza types A and B, its protein products include the viral ribonucleoprotein (vRNP) complex, the matrix proteins (M1 and M2), non-structural proteins (NS1 and NEP), neuraminidase (NA), haemagglutinate (HA) and the polymerase subunits (PB1, PB2 and PA) which form the RdRP. Though siRNAs have been tested preclinically against in influenza virus infection models (see review by Mehta et al., [110]), non-LNP delivery modalities have been employed so far. Early work by Tompkins et al., [100] encapsulated siRNAs targeting IAV nucleoprotein (NP) and PA genes in the commercially available Oligofectamine, resulted in significantly reduced lung virus titres in infected mice and protected animals from lethal challenge. Importantly, siRNA treatment was broadly effective against both pathogenic avian and human IAVs. However, we have yet to see a preclinical study that uses synthesized LNPs for anti-influenza virus siRNA delivery.

#### SARS-CoV-2

SARS-CoV-2 is a betacoronavirus and a positive-sense single-stranded RNA virus (Class IV). It is now clear that current COVID-19 antivirals are losing effectiveness against the highly evolving variants appearing in populations [111]. This makes the programmable approach of using siRNAs to target SARS-CoV-2 attractive. Early in the pandemic, seminal work by Idris et al., [25] showed that siRNAs targeting against various conserved sites in the SARS-CoV-2 genome (5′untranslated region (5′UTR) and conserved stem and the helicase region) encapsulated in LNP formulations composed of DOTAP with the ionizable lipid DLin-MC3-DMA (dmLNP), led to an almost complete ablation of lung viral infection and clinical improvement when administered as an intravenous infusion. A later study then followed this up using the same LNP formulation for intranasal administration of siRNA-LNP and found that siRNA targeting the 5′UTR reduced lung viral loads [27]. A further extension of this study then designed a more potent and ultra conserved anti-SARS-CoV-2 siRNA targeting the helicase region [26]. Here, they showed that dmLNP formulated siRNAs intranasally delivered into SARS-CoV-2 infected mice reduced both lung and nasal viral loads. This demonstrated the first anti-COVID-19 therapy that can target both the upper and lower respiratory regions.

#### Respiratory syncytial virus

Respiratory syncytial virus (RSV) is a negative-sense single-stranded RNA virus (Class V) belonging to the paramyxoviridae family. A recently approved antiviral therapy for RSV infection is Nirsevimab (Beyfortus), an RSV fusion (F) protein-targeted monoclonal antibody, which are only for use in newborns and infants [112]. Other off-label treatment such as palivizumab and ribavirin have also been used for the management of RSV infections but with limited effectiveness [113]. Anti-RSV siRNAs have been tested in vivo in the past [114, 115], none have utilized LNPs as a delivery vehicle. siRNA therapies against RSV have been attempted, ALN-RSV01 is a nasal spray formulation consisting of a single naked siRNA directed against RSV nucleocapsid (N) protein gene [116]. Recent work by Supramaniam et al., [27] used an siRNA targeting the P gene formulated in a stealth lipid nanoparticle (sLNP) was tested prophylactically via both the intranasal and intravenous routes. Both interventions reduced lung viral load in RSV infected mice.

### Future perspectives

In the current health context, the incidence of viral infections and co-infections, such as the concurrent presence of viral hepatitis and HIV [117] and SARS-CoV-2 co-infections with RSV and IAV [118], poses significant challenges to both diagnosis and therapeutic strategies. Alarmingly, IAV co-infections have been documented to augment severity and elevate mortality rates among COVID-19 patients, highlighting the intricate consequences of viral co-infections on clinical outcomes [119]. siRNA-LNP therapies offer a significant advancement, providing a precise approach to simultaneously target multiple viral genomes, either of the same or different virus, using just a single formulation [86, 120]. This strategy presents new possibilities for managing viral co-infections, potentially improving patient outcomes significantly.

Whilst siRNA therapies may be used as an antiviral agent, its innate ability for gene silencing allows its application to use for other disease states. This includes the treatment for ocular conditions, which was halted in phase III trials (tivanisiran, clinical trial #NCT04819269) due to off-target and immunostimulatory effects. Perhaps such an issue may be mitigated with improved delivery platforms, although targeting the eye may prove difficult. Similarly, brain delivery is also another challenging platform. However LNPs have been shown to deliver to the CNS for ischemic brain injury [121] and glioblastoma [122]. Furthermore, the role of siRNA-LNPs in oncology for breast cancer, acute myeloid leukemia (AML), and liver conditions, illustrates its broad applicability [123]. In cancer treatment, multi-drug resistance (MDR) presents a significant challenge to chemotherapy, often resulting in treatment failure and recurrence [124]. siRNA-LNPs present a novel approach by specifically silencing posttranscriptional genes associated with MDR, thereby offering a potential strategy to overcome this challenge.

Despite advances in antiviral siRNA-LNP formulations, the full clinical application of RNAi faces challenges: mitigating RNA-induced cytotoxicity and immunostimulation, enhancing molecular stability, and preventing viral resistance. Furthermore, enhancing organ-specific delivery remains a critical research focus. By fine-tuning LNP formulations, targeted organ delivery becomes possible, optimizing efficacy and minimizing systemic side effects. This organ-targeted approach is crucial for diseases affecting specific organs, such as lung-targeted viral infections. An important issue in clinical translation is the potential to scale up production. Methods of rapid-mixing through different production methods of LNP-siRNA formulations are improving, with several strategies to increase output from microfluidic setups [125]. Advances in such targeted delivery methods are pivotal, potentially revolutionizing siRNA therapeutics towards personalized medicine and markedly improving patient care, whilst simultaneously allowing researchers to investigate the antiviral effects of siRNA.

Whether or not LNPs continue to be a top contender as a non-viral delivery system for nucleic acid therapeutics, other systems exist that has also been successful in the deliver siRNA. One example includes hierarchical mesoporous carbon (MPC) nanomaterials that are derived from the carbonized chitosan (CTS) [48]. Another strategy involves the use of graphene oxide (GO) and the nanocarriers of cell penetrating peptides (CPPs). GO provides a larger surface area and zeta negative potential that enhances the complexing of oligonucleotides [126]. Extracellular vesicles, such as exosomes are also gaining traction, due to them being naturally occurring in biology. Recently, milk-derived exosomes, provided superior structural stability for oral delivery to alleviate inflammatory bowel disease [127]. Finally, niosomes, previously has been used for the delivery of small molecules and macromolecules, spanning to several clinical trials against cancer and fungal infections [128]. However, they share a lot of structural similarities with liposomes and LNPs [129], thus has also been investigated in the delivery of siRNA [130].

## Conclusion

This review outlines the progress in siRNA therapeutics facilitated by LNP delivery, indicating a substantial advancement in antiviral therapy approaches. It discusses the comprehensive efficacy of these therapeutic approaches against a variety of viral pathogens, emphasizing the unique ability of siRNA molecules to precisely target and silence specific viral genes and proteins. This precision in targeting offers an innovative strategy for mitigating viral replication and addressing the drug resistance challenges inherent in traditional small molecule therapies. The development and refinement of LNP platforms, featuring technological enhancements such as ionizable lipids for targeted tissue delivery and structural modifications to improve stability, are imperative for enhancing the efficacy of siRNA-based treatments. Furthermore, the regulatory approval of Onpattro^®^ [9] alongside the success of mRNA vaccines for COVID-19 corroborates the efficacy and utility of LNP-mediated delivery systems in the field of antiviral therapeutics.

However, despite these advances, there remain significant challenges in ensuring LNP formulation stability and optimizing their therapeutic impact while minimizing immune reactions and side effects. In short, this review highlights the critical need for ongoing research to enhance these therapies further and their integration with current treatment regimes, aiming to elevate patient outcomes and contribute to the advancement of antiviral medicine. Importantly, bridging the gap between research and development to clinical translatability may be fast-tracked through the use of studying the use of siRNA as an antiviral agent.

## Data Availability

As this is a review paper, all supporting tables and figures were generated based on previously published research, which is cited throughout the manuscript. Additional details are available from the corresponding author upon reasonable request.
